# Xiroi II, an Evolved ASV Platform for Marine Multirobot Operations

**DOI:** 10.3390/s23010109

**Published:** 2022-12-22

**Authors:** Antoni Martorell-Torres, Eric Guerrero-Font, José Guerrero-Sastre, Gabriel Oliver-Codina

**Affiliations:** Department of Mathematics and Computer Science, University of the Balearic Islands, Ctra. de Valldemossa Km. 7.5, 07122 Palma, Spain

**Keywords:** autonomous surface vehicle, localization, navigation, coordination strategies, marine multirobot systems, marine communications, underwater acoustic communication

## Abstract

In this paper, we present the design, development and a practical use of an Autonomous Surface Vehicle (ASV) as a modular and flexible platform for a large variety of marine tasks including the coordination strategies with other marine robots. This work tackles the integration of an open-source Robot-Operating-System (ROS)-based control architecture that provides the ASV with a wide variety of navigation behaviors. These new ASV capabilities can be used to acquire useful data from the environment to survey, map, and characterize marine habitats. In addition, the ASV is used as a radio frequency relay point between an Autonomous Underwater Vehicle (AUV) and the ground station as well as to enhance the Acoustic Communication Link (ACL) with the AUV. In order to improve the quality of the ACL, a new Marine Multirobot System (MMRS) coordination strategy has been developed that aims to keep both vehicles close to each other. The entire system has been successfully designed, implemented, and tested in real marine environment robotic tasks. The experimental tests show satisfactory results both in ROS-based navigation architecture and the MMRS coordination strategy resulting in a significant improvement of the quality of the ACL.

## 1. Introduction

Currently, the study of the seas and oceans is in a growing stage. These studies range from benthic mapping or alien algae detection to anthropogenic impact monitoring, to name but a few.

The unstoppable demand for this kind of studies and the inherent high risk of the marine environment for human divers, lead to the growing interest in research and development of marine robotic vehicles such as Autonomous Underwater Vehicles (AUV) and Autonomous Surface Vehicles (ASV).

Although researchers have made significant advances in marine robotic vehicles, they have so far been mostly focused on the use of a single vehicle, which has multiple limitations. In contrast, systems with two or more autonomous robots that collaborate to perform a common mission, from now on referenced as Multirobot Systems (MRS), provide several advantages compared to single robot systems, for example: robustness, parallelism, flexibility, scalability, and versatility [1].

This article focuses on describing new capabilities of an ASV, called Xiroi II, initially introduced in [2]. The upgrade of the new vehicle has addressed to the following four objectives.

1. The software architecture must be ROS-based, open-source, and easy to adapt to other ASV’s.
2. The ASV must navigate accurately in order to act as a reliable observation platform for shallow waters benthic habitats.
3. The ASV should act as a relay communication point with the GS and the AUV enabling operations in deeper and more distant coastal areas.
4. The vehicle must be suitable for use as part of a MMRS and implement a coordination strategy to improve the quality of the ACL.

In reference to the first objective, we aim for this work to be a practical guide to facilitate the integration of an open-source control architecture for ASVs. For this purpose, an extensive explanation based on hardware and software implementation and adaptation to an ASV have been carried out.

As for the second objective, thanks to the precise navigation of the ASV, it is able to collect data in shallow water environments, inaccessible to an AUV.

Referring to the third objective, the ASV’s ability to increase the operating distance makes it possible to operate in deeper and more distant environments where acoustic communications are more suitable.

Acoustic communication methods can operate from few meters to several kilometers [3] and have been widely used for decades in diverse underwater applications like oceanography, commercial, search and rescue, offshore industry, and defence [4]. The underwater acoustic channel has some advantages over other technologies, for example, a wide transmission distance range. However, acoustic waves still have many drawbacks including scattering, high delay due to the low propagation speeds, high attenuation, and low bandwidth. These problems depend to a large extent on the distance between the acoustic emitter and the receiver. Thus, the vehicles in a MMRS have to coordinate their movement in order to maintain the communication link between them and ensure a minimum level of quality.

The fourth objective is directly related to the above-mentioned explanations. A novel MMRS coordination strategy has been implemented with the aim to improve the acoustic communication performance between an AUV and an ASV. The coordination strategy have been developed and tested in a real marine environment using two heterogeneous marine vehicles. Finally, an analysis of the communication performance applying this strategy have been carried out. According to this analysis, the distance between the AUV and ASV has a great impact on the communication link and, therefore, it must be take into account in the coordination mechanism.

It is worth mentioning the engineering work carried out and explained in this paper. All software implementations related to the control architecture or the MMRS coordination strategy have been tested and validated in a real marine environment. The results of these experiments show the impact of the MMRS coordination strategy on the system.

The implementations, experiments, and analysis described in this article have been carried out with the Xiroi II ASV, which will be described extensively below in terms of hardware and software architecture. To perform the heterogeneous MMRS, an unit of the commercial AUV *Sparus II* has been used. We refer the reader to [5] for a detailed description of the software and hardware of the Sparus II AUV. The AUV has a secondary role in this article and its presence is justified only because it is part of the MMRS.

Both vehicles, the ASV and the AUV, define the heterogeneous MMRS that will be used to test the effectiveness of the coordination strategy. These vehicles have been endowed with an Ultra Short Base Line (USBL) system, which allows the acoustic communication between them as well as their relative positioning.

There is also a Ground Station (GS) equipped with a Global Positioning System (GPS) receiver that sends positioning corrections to the ASV via a Radio Frequency Link (RFL). Additionally, the ASV is endowed with another GPS and the aforementioned USBL, so acting as a communication relay point between the AUV and the GS. The entire communications system implemented in the MMRS will be described below.

All the software described in this paper has been developed on the open-source framework Robot Operating System (ROS) [6,7]. This framework allows programmers to reuse and join pieces of software developed by other programmers in order to create new functionalities. Thus, over the last years, ROS has become an standard in programming robots.

The paper is structured as follows: the state of the art concerning surface marine vehicles and MMRS is presented in Section 2, Section 3 presents the design of the new ASV platform, Section 4 summarizes the integration of the software architecture, Section 5 exposes a novel MMR coordination strategy, Section 6 covers the ASV navigation and control tests results, and Section 7 analyzes the MMRS tracking strategy and how this strategy improves the ACL. Finally, the conclusions and future work are presented in Section 8.

## 2. Related Work

A great number of ASVs with different capabilities and functionalities was developed, mostly in the last twenty years, arising from academic and research institutions [8]. The current literature provides detailed ASV’s state of the art like [9,10] or, more recently, [11,12]. Regarding research and development, several relevant projects have been reported, carrying out different operations in aquatic scenarios like in [13,14,15].

In terms of platform design, depending the applications and the navigation environment, the developer should pay special care on the stability, the maneuverability, the speed and the safety of the ASV. Platform designs can mainly be divided in monohull [16,17,18] and multihull [8,19,20,21,22] solutions. Looking on current catamaran hull designs, the AERO4RIVER ASV [23] presents a new propulsion system based on aerial thrusters that allows allows navigation in very shallow waters. The pieces of work of [13,24], where the SWATH ASV is described, study the optimal catamaran hull form in order to provide low resistance and excellent station keeping performance in irregular head waves. In [25], the SWAMP ASV was presented highlighting the modularity and the extremely shallow depth navigation using a water pump as a propulsion system. In [26] the NEREUS ASV, manufactured using off-the-shelf components, was presented.

In terms of software architecture, [27,28] presented a solution for marine vehicles implemented as a toolchain that consists of 3 main entities: DUNE onboard software, Neptus command and control software and a common IMC message-based communication protocol. Other interesting work developed in [29] presents a ROS-based control architecture for AUVs named COLA2 (Component-Oriented Layer-Based Control Architecture). This software architecture implements three layers: reactive, control, and mission. The reactive layer manages the sensors and actuators at a low level. The control layer is responsible for generating the guidance orders, while the mission layer is responsible for instantiating the appropriate vehicle’s behaviour to achieve the mission [5]. The Xiroi II software architecture is based on COLA2, although several changes that will be described later have been introduced.

As far as Marine Multirobot Systems (MMRS) are concerned, in recent years, an increasing number these systems have been presented in the literature with the aim of discovering, exploring, and analyzing regions previously inaccessible to conventional systems in marine environments [30,31,32,33]. To take advantage of the MMRS benefits, robots must execute coordination strategies that, in general, require a certain degree of communication between them. How, when, and what robots should communicate remains an open question in MRS and especially in scenarios with severe communication constraints, such as the maritime environments discussed in this paper.

For all the above mentioned, the focus in recent years has been on coordination strategies using ASVs and AUVs [33,34,35,36,37,38,39]. In [34], an AUV is required to inspect several goals while staying within the communication range of an ASV. Authors in [35] presents an ASV able to follow an AUV without prior knowledge of the AUV’s actual position. The work done by [37] implements an active acoustic communication protocol that prioritize the AUV positioning rate. It should be noted that none of them makes a study on the quality of the acoustic signal.

## 3. Hardware Design

In this Section, we describe the hardware design of the Xiroi II ASV, including mechanical issues, electronics design, sensors, and communication mechanisms.

**Figure 1:**
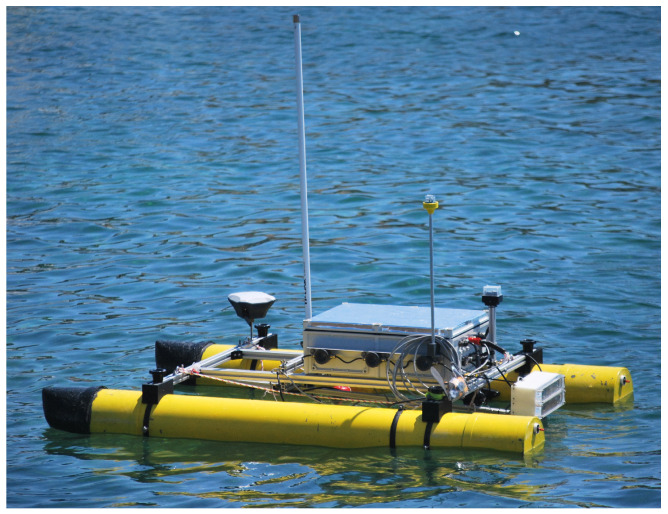
Xiroi II ASV morphology.

The robotic platform has been designed to operate in coastal waters and taking into account that its main tasks will be to operate in shallow waters and to relay communications between an AUV and a GS. Thus, a two-hull catamaran solution has been chosen to achieve a robust, roll-stable, and redundant structure [40]. Other advantages of the catamaran include its payload capacity, shallow draft, deck accessibility, and modularity. The modularity has other important benefits as it allows the power system, the electronics, the sensors and the actuators to be separated, not forgetting that the vehicle can be easily disassembled to transport, check, and repair it. The Figure 1 shows the morphology of the Xiroi II ASV.

Due to the modularity and large payload capacity, the vehicle can be equipped with different sensors such as stereo cameras, sonars, or biochemical sensors. In addition, the vehicle equips an USBL when it is intended to be used as an acoustic communication relay point between an AUV and a GS. Using this ASV functionality, the operational distance between the AUV and the GS can be increased.

The GS allows controlling and monitoring the ASV operation through a radio frequency connection. In addition, the GS also includes a GPS base that receives position corrections and sends them to the GPS rover on the ASV. Details about that functionality are given in the following Subsections.

### 3.1. Vehicle Design

Xiroi II ASV has been designed choosing off-the-shelf components that can be easily replaced or even improved and making the vehicle easy to assemble and disassemble to save deploying time. The vehicle frame has been manufactured using extruded aluminum profiles and the hulls are made with Polyvinyl Chloride (PVC) pipes. The electronic components, including the computer and the batteries are installed in a watertight box mounted just above the hulls. Table 1 summarizes the main ASV specifications. The vehicle is equipped with two Blue Robotics T-200 thrusters, the computational system is composed by a Intel Core i7 PC running Ununtu 18.04 and ROS Melodic, and the whole system is powered by a Lithium-Ion battery. The vehicle is 1.75 m long and 1 m wide, with a weight of 45.8 kg and a payload capacity of about 20 kg.

### 3.2. Electronics

**Figure 2:**
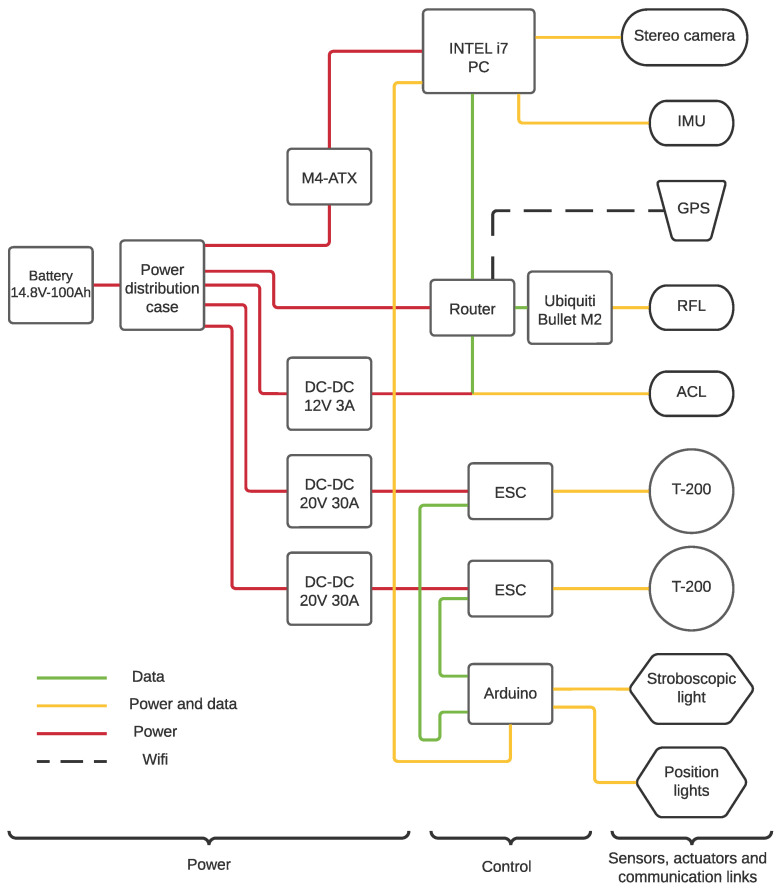
Xiroi II onboard electronics. Red lines represents the power wires, green lines represents the data wires, yellow lines represents data and power wires, and black stripped lines represents a WiFi connection.

The onboard electronics is decomposed into three main layers: the power layer, the control layer, and the sensors, actuators, and communication links layer. Figure 2 shows the different layers and devices that make up the electronics system.

- Power Layer

The power layer includes the battery pack and a series of DC-DC voltage converters in order to obtain the required input voltage for the different devices such as the thrusters, the router, or the main PC.

The battery pack is manufactured using a ion-lithium rechargeable cell battery as a main element of the power supply system. Packing the cells properly, we obtain the following battery pack characteristics shown in Table 2.

In terms of energy conversion, two dedicated DC/DC converters power the Electronic Speed Controllers (ESC) that controls the T200 s actuators at 20 V up to 30 A. Furthermore, another DC/DC energy converter is used to power the peripherals such as the onboard router. The M4-ATX board provides the energy to the PC.

- Control Layer

The control layer contains the principal process and control units as the onboard PC and an Arduino microcontroller board in addition to a router acting as a central communication point.

The Arduino microcontroller board is used to convert the computed Revolutions Per Minute (RPM) requested by the architecture to Pulse Width Modulation (PWM) signals to the ESC’s. In terms of computational capacity, the ASV onboard computer consists of an Intel-i7 processor with 16 Gb of RAM. The onboard computer, which handles with the software architecture, uses two storage disks, a Solid State Disk (SSD) as a process unit and a Hard Disk Drive (HDD) as a data logger unit. The computer specifications can be seen in Table 3.

- Sensors, Actuators, and Communication Links Layer

The third layer includes the sensors such as the Inertial Measurement Unit (IMU) and the GPS, the thrusters, the Acoustic Communication Link (ACL), the Radio Frequency Link (RFL) communication, the stroboscopic light, and the position lights.

The Emlid Reach RS GPS and the Memsense Nano IMU are the main navigation sensors of the ASV. The GPS uses a Global Navigation Satellite System (GNSS) based on GPS and Global’naya Navigatsionnaya Sputnikovaya Sistema (GLONASS). This device uses different corrections sources as Differential Global Positioning System (DGPS), Real Time Kinematic (RTK) or Networked Transport of RTCM Internet Protocol (NTRIP) to improve data positioning signal. The GPS system is composed by two GPS units, the GPS base and the rover. The GPS base is fixed to the GS, and the GPS rover is placed on the ASV. The GPS base receives NTRIP corrections and transmits RTK corrections to the GPS rover in order to improve the precision.

The Memsense Nano IMU is used to provide the linear acceleration, rotational speed, and heading data. The device includes a magnetometer, which provides an absolute estimate orientation of the vehicle. In order to avoid magnetic perturbances with the computer or other electrical devices, the IMU is placed into a watertight enclosure outside of the electronics case.

**Figure 3:**
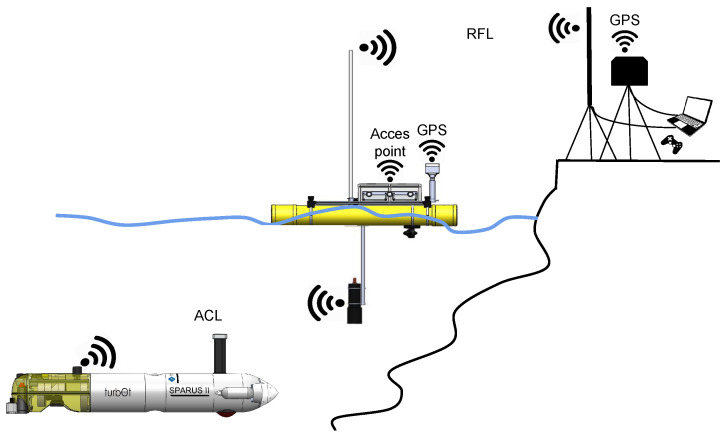
Communication scheme.

Several communication links assure a reliable and stable data connection between the vehicle and the GS. On the one hand, the GPS base, placed at the GS, transmits the corrected positions to the GS portable computer (PC) through WiFi. This PC is connected to the RFL antenna, which links the GS with the ASV. The RFL is made up of an Ubiquiti Bullet B-DB-AC device and an omnidirectional radio-frequency antenna which ensures a long communication range up to 3 km. Focusing on the vehicle, the rover GPS transmits the position information through the WiFi link to the onboard PC. This computer is equipped with a router which acts as an access point in order to receive information via WiFi. In the same way as in the GS, the onboard RFL is connected to the onboard ASV PC. Moreover, the Xiroi II ASV can establish an ACL with the AUV so that both vehicles can share information and the former has the possibility to manage the submarine through this link. The overview communication scheme can be seen in Figure 3.

In terms of propulsion system, two Blue Robotics T200 brushless thrusters move the ASV. This differential drive configuration allows the vehicle to be properly navigated and manoeuvred. Powering the motors at 20 V up to 30 A provides the required navigation speed.

## 4. Software Architecture

As mentioned above, the software architecture has been build using ROS since it offers many advantages:

- Software portability, reuse, and sharing, thus improving its maintainability.
- Avoids crosscompiling and strengthens the data integrity of the whole system.
- It enables a pure distributed software and applications.

In this Section, we describe the integration of the COLA2 ROS-based software architecture in the Xiroi II ASV platform. Some COLA2 source packages have been adapted or replaced in order to adjust the architecture to the new ASV characteristics. The platform has been programmed in C++ and Python using ROS Melodic under Ubuntu 18.04. The xiroi_stack [41] repository hosts the required packages to run the COLA2 architecture over the Xiroi II ASV platform. All the specific configurations linked with the following subsections are hosted in a config folder located into the cola2_xiroi package included into the xiroi_stack Github repository.

**Figure 4:**
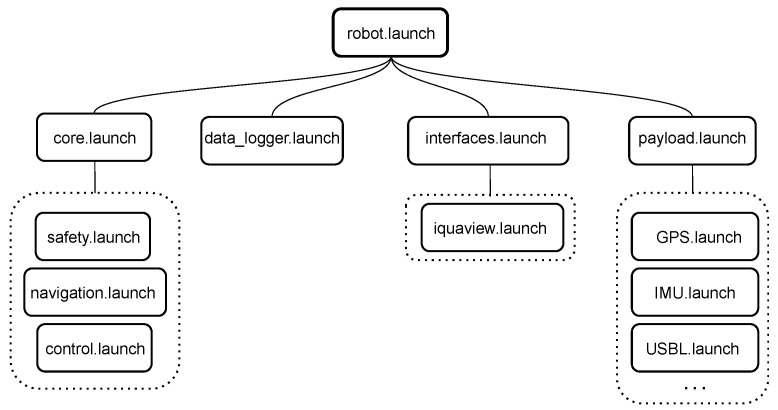
Principal architecture launch files.

The software is structured in a pyramidal way, where the xiroi_stack repository hosts all the packages involved in the architecture, such as the sensor drivers or the navigation and control modules. The cola2_xiroi package, included into the xiroi_stack, allocates all the specific configurations for the Xiroi II ASV. This package has the main function of managing the different parts of the xiroi_stack architecture: control, navigation, sensors, safety, and interfaces. All these parts are managed using launch files, which are scripts having the capacity to simultaneously activate launch files and/or ROS nodes, understanding a ROS node as a piece of code. As can be seen in Figure 4, the robot.launch handles all the principal parts of the vehicle architecture.

The description of the functionality of the main launch files is as follows. The core.launch manages the safety, navigation, and control strategies implemented in the COLA2 architecture. These strategies have been tuned specifically in order to obtain an accurate navigation and control behaviors.

The payload.launch is in charge of the sensor drivers like the IMU, the GPS, the stereo cameras or the USBL head.

The interfaces.launch launches the graphical user interface (GUI) called Iquaview [42]. Iquaview GUI is an original part of the COLA2 architecture that helps the user to define the different navigation strategies, which will be introduced in Section 4.3, as well as control and monitor the vehicle during the navigation process. More information on the installation and basic use of Iquaview is available at [43].

Finally, the data_logger.launch handles data recording by selecting the essential information to minimize the size of the stored files.

### 4.1. Localization

The original COLA2 localization layer integrates the specific drivers for all the possible AUV navigation sensors: IMU, GPS, Doppler Velocity Logger (DVL), USBL, and pressure sensor. Instead of using this localization strategy, the Xiroi II uses the robot_localization package [44], which is more adaptable and versatile. In our system, the navigation sensors are reduced to an IMU and a GPS. In order to obtain the vehicle localization, the GNSS and the IMU data are fused in a standard Extended Kalman Filter (EKF). Before applying this filter, few preprocesses, explained next, are carried out in order to obtain better results.

- Sensor Aggregator

The sensor aggregator node has the main function of ensuring that the IMU and the GPS data provided by the drivers have the sufficient quality to obtain a correct estimation of the vehicle position. This node is also in charge of adapting the GPS and IMU data to the specific format that the Robot Localization (RL) package requires.

- IMU filter Madgwick

Prior to the EKF, the IMU data is filtered using Madgwick’s IMU filter [45]. The Madgwick’s filter allows to fuse the angular velocities, accelerations, and magnetic field data extracted from the IMU driver in order to obtain a more reliable orientation estimation.

- Robot Localization filter

The RL package is based on a standard EKF, where the inputs used are latitude *x* and longitude *y* from the GPS and the orientation γ and angular velocity $\dot{\gamma }$ from the IMU. These reading are used to update the EKF, whose state vector is formed by $\left(x,y,\gamma ,\dot{x},\dot{y},\dot{\gamma },\ddot{x},\ddot{y}\right)$, being (x,y) the vehicle global position, γ the vehicle heading, $\dot{x},\dot{y}$ the vehicle lineal velocity, and $\ddot{x},\ddot{y}$ the lineal acceleration.

The RL package has specific parameters to define how the data are treated before being fused with the core filters in order to adjust the prediction to the vehicle characteristics. These parameters define the operating mode for RL. The main parameters are the coordinate frames: world_ned, odom, and base_link. The base_link frame is the coordinate frame that is affixed to the robot. The robot’s position in the odom frame will drift over time but is accurate in the short term and should be continuous. The world_ned frame, like the odom frame, is a world-fixed coordinate frame, and while it contains the most globally accurate robot estimate position, it is subject to discrete jumps. More detailed explanations about the RL package and the coordinate frames conventions can be found in [46,47].

### 4.2. Control

The COLA2 control layer is based on a cascaded scheme in which a pose controller node, a velocity controller node, and a thruster node are used to control the system. This topology offers several options to control the vehicle using a force, a velocity, or a pose ROS topic.

The Xiroi II ASV can be actuated in 2 degrees of freedom (DoF), surge and yaw. In order to adjust the original COLA2 controller to this system, some parameters have been configured to satisfy the new requirements.

- Thruster Control Matrix

The thruster control matrix (TCM) specifies how each thruster contributes to the force or torque generated in each axis of the robot. The columns represent the left and right thrusters, respectively, and the rows represent surge, sway, heave, roll, pitch, and yaw respectively. The yaw parameter is related to the distance between the thruster and the center of the vehicle. In our case, the thrusters are 45cm apart from the longitudinal axis of the vehicle. Then, the resulting TCM can be seen in the following matrix (1)

(1) $$TCM:\left[\begin{array}{cc} 1.0 & 1.0\\ 0.0 & 0.0\\ 0.0 & 0.0\\ 0.0 & 0.0\\ 0.0 & 0.0\\ -0.45 & 0.45 \end{array}\right]$$

- Thrusters characterization

Another important point that has to be taken into account in the control module is the thruster characterization models. These parameters characterize both thrusters individually in forward and reverse mode. The controller module uses this information to match the speed to the propellers characterization curves. The Xiroi II ASV uses a differential drive navigation configuration in which the propulsion system is provided by two Blue Robotics T-200 thrusters [48] powered at 20 V. The characterization of the thrusters was obtained through a series of experiments in which the ASV was kept static attached to a dynamometer while each thruster was forced to rotate at different speeds in both forward and reverse directions. The experimental data obtained were plotted on a graph where the X-axis represents the force in Newtons and the Y-axis the Pulse-width Modulation (PWM) applied. Finally, the characteristic curve of each thruster is obtained by fitting a polynomial function on the data generated by each one of them.

### 4.3. Navigation

The COLA2 software architecture implements several navigation strategies that can be sent to the ASV through the Iquaview GUI described in [42]. Iquaview allows to create and define the main parameters of each strategy as well as monitor the ASV during its navigation. As explained below, each strategy has its own navigation control algorithm, the pilot.yaml configuration file allocates the control parameters that defines these navigation control algorithm behaviors at low level. Next, the four main navigation strategies, implemented in COLA2, are described.

- Goto

The goto behavior is one of that basic guidance primitives implemented in COLA2 that moves the vehicle to a specific target. A fixed velocity is applied to the vehicle to reach the desired point. The goal is assumed to be achieved when the vehicle is at a distance from the destination within a tolerance radius parameter set in the pilot.yaml file.

- Keep position

The keep position behavior has the main function of holding the vehicle in a desired area defined by a tolerance radius parameter. This behavior is a particular case of goto primitive parametrized with a static target point where the vehicle yaw is controlled so that the robot always points toward the target.

- Section

The section navigation behavior is used to follow the line comprised between two waypoints. To that end, a Line of Sight (LOS) with Cross Tracking Error controller [49] is applied to extract a yaw pose request to keep the vehicle on the line, reducing the difference between the desired navigation route and the real one. The section behavior implements a velocity adjustment that depends on the distance from the vehicle to the initial and final waypoints, slowing down at the start and final points and going faster in the middle.

- Mission

Finally, missions are combinations of gotos and sections in order to generate more complex trajectories.

## 5. Marine Multirobot Coordination

Coordination and Cooperation of Multirobot Systems (MRS) are widely studied topics in the field of robotics [50,51,52,53]. As can be seen from the literature cited above, the MRS coordination strategies share many challenges. Some of these common issues, as defined in [53], are: the optimal control of each individual robot; the task allocation; the communication between the different agents of the MRS, among others. According to [54], one of the most important aspects to achieve a good coordination is based on ensuring adequate communication between the components of the system. Therefore, one of the main objectives of the ASV Xiroi II is to carry a USBL transceiver head system to improve the quality of the ACL. The following subsections describe a new MMRS coordination strategy based on tracking and navigation coordination to ensure the quality of the communication link between both vehicles.

### 5.1. AUV Tracking Strategy

The tracking strategy aims to keep the ASV as close as possible to the AUV in order to reduce the distance between the acoustic transponder and transceiver while maintaining adequate safety measures to avoid collisions.

**Figure 5:**
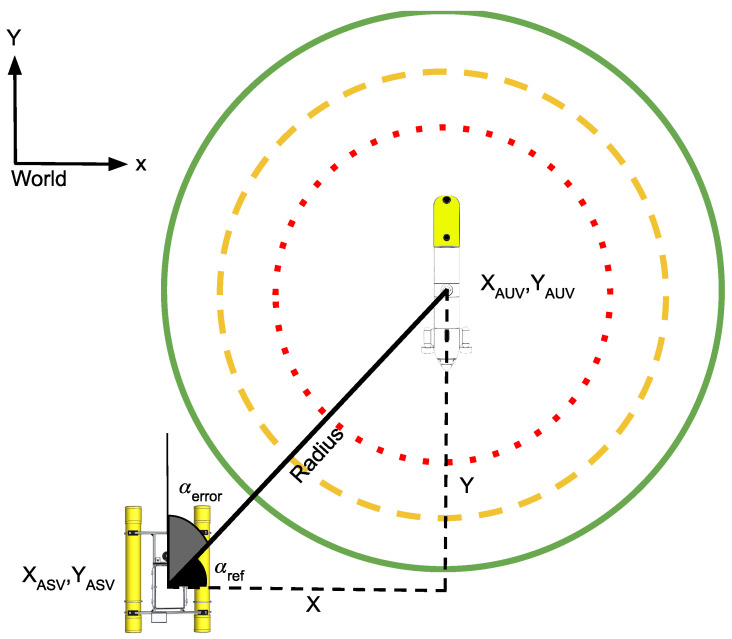
$X_{AUV}$, $Y_{AUV}$, and $X_{ASV}$, $Y_{ASV}$ represent the AUV and the ASV positions, respectively referenced to the world NED reference system. The red dotted circle represents the repulsion radius, the orange dashed circle represents the adrift radius, and the green circle represents the tracking radius.

Figure 5 shows the scheme defined by the tracking, adrift, and repulsion strategies depending on the distance between the ASV and the AUV measured in (X,Y). As can be seen in this figure, three regions (green, orange, and red circles centred on the AUV coordinates) have been defined in which the ASV must apply a different movement strategy (tracking, drifting, or repulsion, respectively) in order to maintain the communication link between the vehicles as best as possible, avoiding a collision. The output of the tracking strategy is the ASV velocity vector ($\dot{x}$, $\dot{y}$, $\dot{\gamma }$). These values are sent to the ASV controller, which translates them to the corresponding ASV movements. The basic principles that apply to this new ASV navigation behavior are:

1. The ASV must keep close to the AUV according to the predefined adrift radius.
2. The ASV must apply a safety repulsion behavior in case it reaches the repulsion radius.
3. In order to minimize acoustic perturbances between the ASV thrusters and the ACL, the ASV will disable the thrusters in the area between the adrift radius and the repulsion radius.

The first and most restrictive state is the repulsion radius, in which the ASV must not remain. This strategy tries to avoid any risk of collision between the AUV and the ASV. For this purpose, if the AUV trajectory, projected on the (X,Y) plane, points to the position of the ASV, the ASV will move perpendicular to this trajectory. This strategy is applied even when the AUV is submerged, allowing it to surface at any time without danger of collision. The first step to be applied in this state is to extract the slope of a straight line joining the positions of the ASV and the AUV, represented in Equation (2).

(2) $$\begin{array}{ccc} m & = & \frac{Y_{AUV}-Y_{ASV}}{X_{AUV}-X_{ASV}} \end{array}$$

Then, the equation of the perpendicular line between the ASV and the AUV that passes through the ASV position is given by (3) at step i:

(3) $$\begin{array}{c} Y_{i}=-\frac{1}{m}\cdot \left(X_{i}-X_{ASV}\right)+Y_{ASV} \end{array}$$

where $X_{i}$ is obtained using the following rules depending of the $X_{AUV}$ and $X_{ASV}$ position:

(4) $$X_{i}=\left\{\begin{array}{c} X_{i-1}-\Delta \;\mathrm{if}\;X_{AUV}>X_{ASV}\\ X_{i-1}+\Delta \;\mathrm{otherwise} \end{array}\right.$$

**Figure 6:**
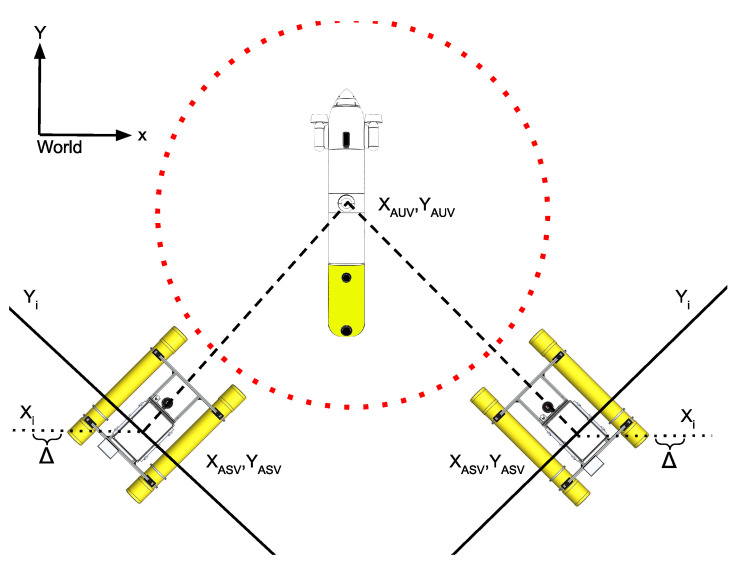
Red dotted line represents the repulsion radius, and black line represents the perpendicular line between the ASV and the AUV, $Y_{i}$. Finally the black dotted line represents the $X_{i}$ values where the Δ is the step increment. Note that the reference coordinates has been placed at the top left-hand side of the image.

Figure 6 shows graphically the logical process applied to design the repulsion strategy. In the example, where the ASV is on the right side of the image, the $X_{ASV}$ is larger than the $X_{AUV}$; then, the $X_{i}$ must increase generating a tangent trajectory to the right defined by the $Y_{i}$ equation. Similarly, if the ASV is located on the left side of the image, the $X_{ASV}$ is smaller than the $X_{AUV}$; then, the $X_{i}$ must decrease, generating a tangent trajectory to the left side defined by the $Y_{i}$ equation. In both cases, the step value Δ was set to 1 m in order to decrease or increase, respectively. This strategy ensures that the ASV effectively avoids the trajectory of the AUV, thus preventing the risk of collision.

Once the safe position $\left(X_{i},Y_{i}\right)$ has been obtained, the output velocities required to achieve a target point can be calculated, as shown in the following expressions. $\alpha_{ref}$ is the target angle for the vehicle to move to the goal safe position and $\alpha_{error}$ stands for the angle between the ASV orientation and the desired angle $\alpha_{ref}$. In Equation (6), the atan2 function is applied to obtain the smallest angle between both positions. The repulsion strategy should be fast enough to avoid collisions, so a constant linear (*v*) and angular (ω) velocities are set. Finally, in Equations (7)–(9), the computed linear and angular velocities are extracted.

(5) $$\begin{array}{ccc} \alpha_{ref} & = & atan2(Y_{i},X_{i})\; \end{array}$$

(6) $$\alpha_{error}=atan2(sin\left(\alpha_{ref}-yaw_{ASV}\right),cos\left(\alpha_{ref}-yaw_{ASV}\right)\;$$

(7) $$\begin{array}{c} angular\_velocity=\dot{\gamma } \end{array}$$

(8) $$\begin{array}{c} \dot{x}=v\cdot cos\left(\alpha_{error}\right) \end{array}$$

(9) $$\begin{array}{c} \dot{y}=v\cdot sin\left(\alpha_{error}\right) \end{array}$$

The second state covers the distance between the repulsion radius and the adrift radius. In this area, the ASV disables its thrusters to avoid acoustic perturbances and waits for the AUV movements.

The third and final state includes the reminder of the space. Within this area, the ASV applies a tracking behavior, going at the maximum speed while outside the tracking radius and adjusting its speed between the predefined tracking and adrift radius to avoid continuous entry and exit within the adrift radius. Equation (10) normalizes the velocity depending on the distance between the ASV and the predefined adrift radius. In Equations (11) and (12), *v* and ω velocities are adjusted, depending in the first case on the v_adjustment and in the second on the $\alpha_{error}$. Note that the $\alpha_{error}$ can be extracted the same way as in Equation (6). Finally, the output velocities are obtained from Equations (5)–(9), taking into account the new values of the adjusted velocities *v*, ω and $\alpha_{ref}$.

(10) $$\begin{array}{ccc} v\_adjustment & = & \frac{radius-adrift\_radius}{tracking\_radius-adrift\_radius} \end{array}$$

(11) $$\begin{array}{ccc} v & = & v\_adjustment\cdot v \end{array}$$

(12) $$\begin{array}{ccc} \omega & = & \alpha_{error}\cdot \omega \end{array}$$

## 6. Platform Tests

Several tests have been carried out to check the effectiveness of the integration of the COLA2 software architecture and the performance of the new ASV MMRS strategies. These tests were conducted in Cala Comtesa (39°32′05.2′′ N, 2°35′23.2′′ E), a coastal area with shallow water and calm sea conditions with sandy sea bottom mixed with Posidonia Oceanica Seagrass located in the bay of Palma de Mallorca, Spain.

### 6.1. Navigation Tests

In order to validate the above mentioned ASV behaviors, different navigation strategies, such as keep position, goto, section, or survey, have been tested. All of them have been implemented in the COLA2 architecture. Furthermore, the Iquaview GUI [42] is used to program and send the specific navigation strategies to the ASV.

- Keep position

**Figure 7:**
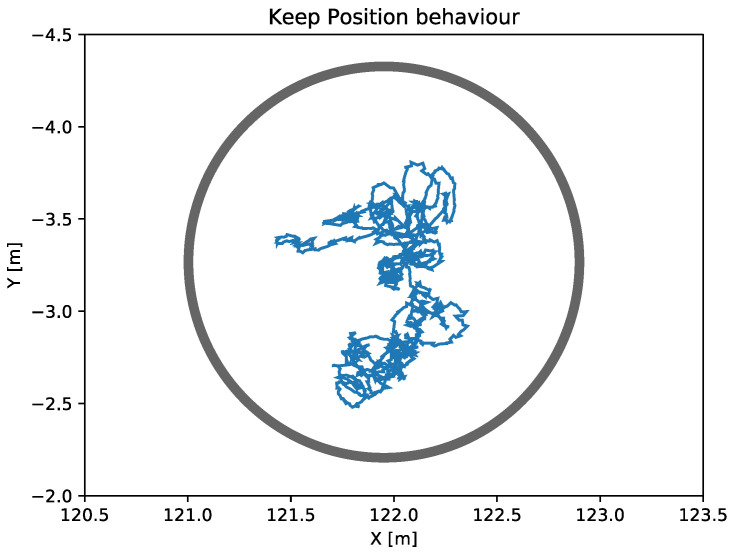
The blue line represents the displacement of the vehicle caused by the waves and currents for about 270 s applying a keep position behavior. The grey circle represents the tolerance radius set in the pilot.yaml that defines the area in which the vehicle should remain.

The main objective of the keep position behavior is to maintain the vehicle into a desired area defined by a tolerance radius parameter. Figure 7 represents a real test of the keep position behavior where the ASV was applying this navigation behavior for more than 4 min while remaining within the defined tolerance radius at all times.

- Goto

**Figure 8:**
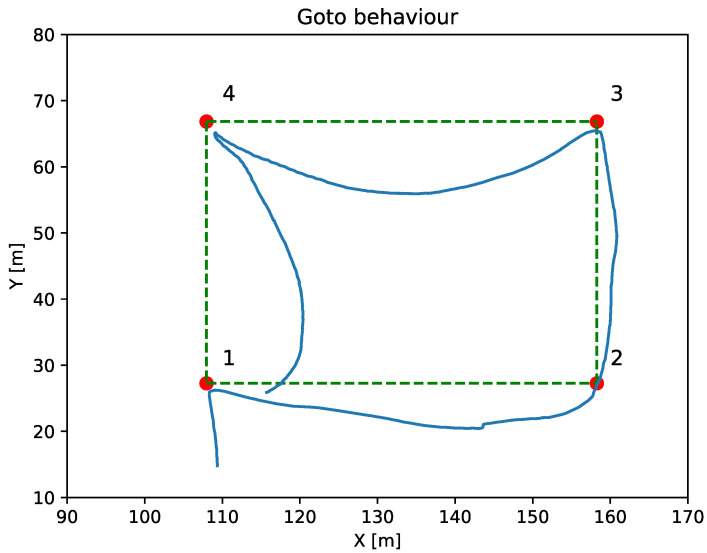
The red dots represent the goto desired waypoints. The blue line represents the vehicle trajectory and the green dashed line represents the ideal navigation route.

The goto behavior moves the vehicle to a specific point, whether it is the target or a waypoint. Figure 8 shows a goto mission composed by four waypoints. This navigation behavior does not apply the LOS controller, causing, as shown in the figure, a significant difference between the actual navigation path and the ideal navigation one, even though the vehicle reaches the mission waypoints.

- Section

**Figure 9:**
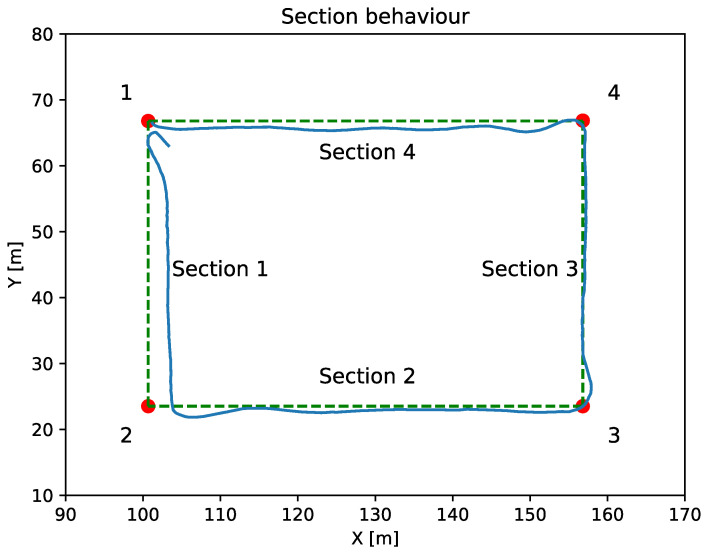
The green dashed line represents the programmed sections comprised between the waypoints represented as red dots. The blue line represents the vehicle route.

The section navigation behavior is used to follow the line comprised between two waypoints. Figure 9 shows the results obtained by applying the section navigation behavior. As can be seen, in this case where a LOS navigation controller is applied, the error between the programmed and actual routes has been substantially reduced.

- Mission

**Figure 10:**
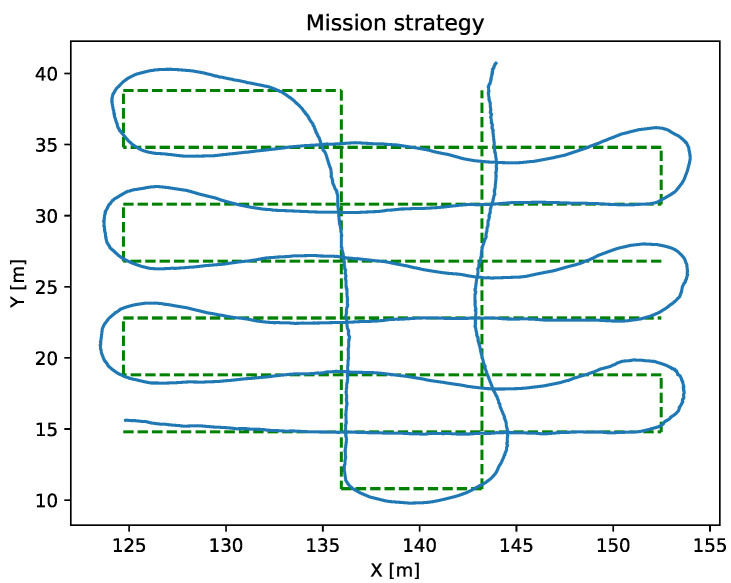
The green dashed line represents the programmed mission path. The blue line shows the navigation route achieved by the ASV.

Missions are combinations of gotos and sections in order to generate more complex trajectories. Figure 10 shows a mission performed using section strategies.

### 6.2. Control Tests

As mentioned above, the control of the ASV depends on the position and velocity controllers. The following tests show the results obtained once the controllers are adjusted to obtain the best results.

- Position controller test

**Figure 11:**
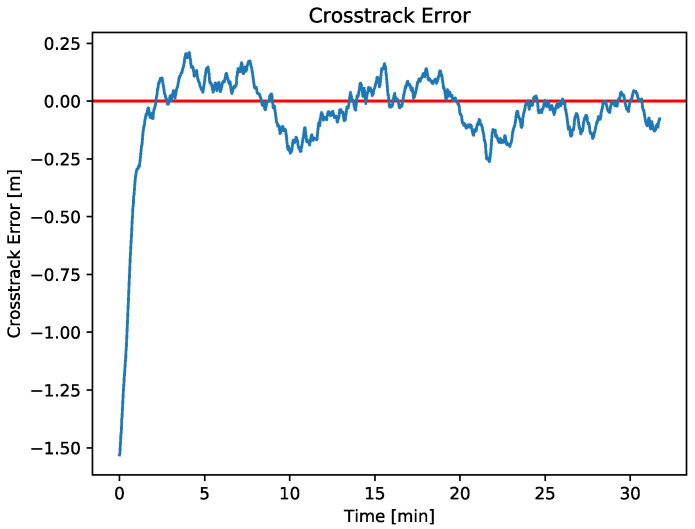
The red line represents the desired 0 crosstrack error. The blue line represents the evolution of the crosstrack error throughout a mission. The sample mean between minutes 2 and 31 is −0.017 and the standard deviation is 0.09.

The LOS algorithm controller defines how the ASV adjusts its movements to a predefined path. To perform the LOS test, a predefined section mission is sent to the ASV through Iquaview GUI. Figure 11 shows the ASV distance crosstrack error between the real path versus the desired path. As can be seen, the maximum error is less than 0.25 m, which is a very low positional error considering that the ASV endured the disturbances of sea currents, wind, and waves.

- Velocity controller test

**Figure 12:**
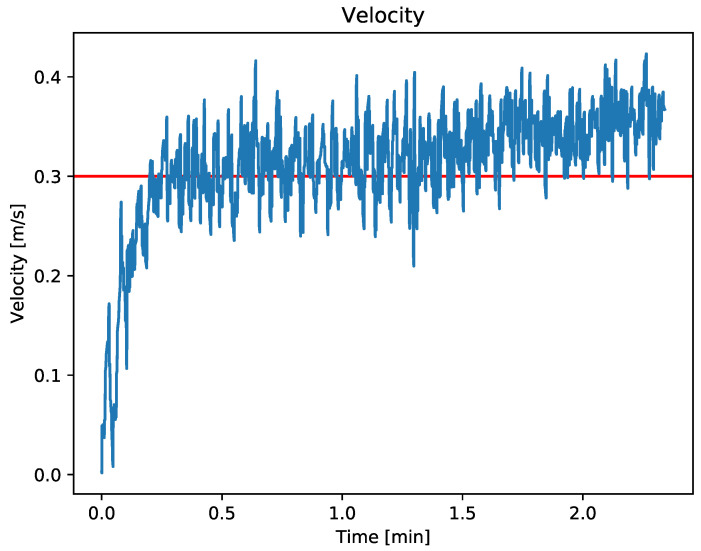
The red line represents the target speed set at 0.3 m/s. The blue line depicts the actual speed of the vehicle during the test. The sample mean between minutes 0.3 and 2.3 is 0.313 and the standard deviation is 0.058.

To perform the velocity controller test, a goto strategy with a predefined velocity was sent to the ASV. Figure 12 shows the ASV velocity along the mission over the time. As can be seen in the figure, the ASV is able to maintain the predefined speed of 0.3 m/s, despite the disturbances caused by wind and waves.

## 7. MMRS Tracking Strategy Tests

The MMRS tracking strategy has been developed with the objective of keeping the ASV as close as possible to the AUV to ensure a more reliable ACL, while avoiding collisions between the vehicles. Some recent studies on coordination of MMRS with a similar aim can be found in the literature. See [34,35,37,55,56,57] for example. In contrast to the works cited above, the present study, in addition to analyzing a real coordination strategy from a navigation point of view, also provides an assessment of how this strategy improves the ACL in terms of the acoustic positioning frequency reception. To assess the effectiveness of the coordination strategy, both the ASV tracking and its impact on the ACL quality have been analyzed.

### 7.1. Navigation Study

In this Subsection, a navigation study has been carried out focused on the evolution of the distance between the ASV and the AUV as well as on the speed applied by the ASV during this process. During this study, the AUV performed a mission where the ASV, acting as a servant, applied the MMRS coordination strategy to track the AUV with the aim to keep as close as possible to the AUV while maintaining adequate safety measures to avoid collisions.

**Figure 13:**
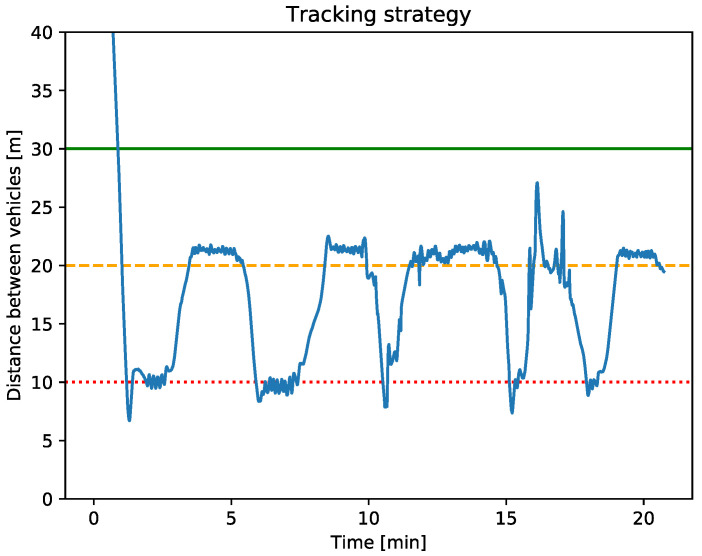
The blue line represents the (X, Y) distance between the AUV and the ASV in meters. The red dotted line represents the repulsion radius (10 m), the orange dashed line represents the adrift radius (20 m), and finally, the green line represents the tracking radius (30 m).

**Figure 14:**
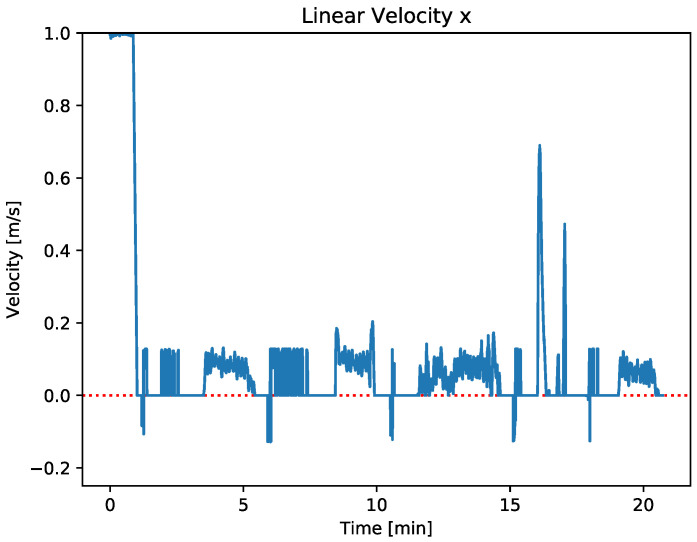
The blue line represents the ASV linear normalized speed $\dot{x}$ extracted from the tracking algorithm, being 1 the maximum and −1 the minimum.

In order to analyze the navigation performance of the strategy, it is essential to compare both Figure 13 and Figure 14.

Figure 13 shows the distance between vehicles obtained while the tracking coordination strategy have been applied where the three behaviors: repulsion, adrift, and tracking, described in Section 5 are applied. On the other hand, Figure 14 represents the linear speed $\dot{x}$ over the time of the Xiroi II ASV. This speed represents the ASV forward and backward movements and how the vehicle adjusts them depending on the distance to the AUV.

The Figure 13 shows the data obtained while the ASV applied the three behaviors: repulsion, adrift, and tracking, described in Section 5.

Focusing on Figure 13, it can be observed that the ASV tends to stay close to the adrift radius. For example, from approximately minute 3 to 5.5 or from minute 12 to 14, the vehicle adjusts its speed to follow the AUV keeping close to the adrift radius. The oscillations present in the stationary parts are due to an abrupt threshold applied between the different radius of the tracking coordination strategy. Nevertheless, the behavior of the ASV and its speed (0.1 m/s) is as expected by the navigation strategy.

Figure 14 shows that, during the first moments of the navigation test, the ASV applies the maximum speed while it is outside the tracking radius. On the other hand, the vehicle adjusts its speed, as its defined in Section 5.1, when the ASV navigates between the tracking radius and the adrift radius. This behavior can be clearly observed during the first moments of the sea trial and between minutes 15 to 20 of the Figure 14, when the ASV adjusts its speed in order to track the AUV.

If the vehicle is between the adrift radius and the repulsion radius, it deactivates the thrusters and keeps drifting.This behavior appears when a transition takes place between the repulsion radius and the adrift radius or vice versa. In that moments, the linear velocity of the ASV is null.

Finally, when the ASV reaches the repulsion radius, it moves away from the AUV to avoid a collision. Focusing on the minutes 1.5, 6, 11, 15, and 18 of the Section 5.1, it can be seen that when the ASV reaches the repulsion radius, the repulsion behavior applies. It is worth noting that in these cases, the ASV applies a negative velocity, as can be seen in Figure 14.

In light of the above experimental results, it can be concluded that the tracking coordination strategy offers the possibility to maintain a predefined distance between two robots in an efficient and collision-avoiding way. Moreover, in all cases, the vehicles show the expected behavior.

### 7.2. Acoustic Communication Study

**Figure 15:**
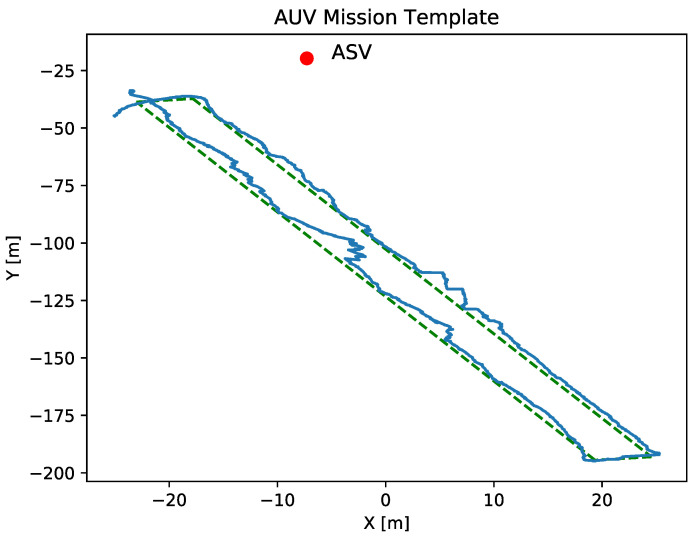
The green dashed line represents the programmed transect test bench for the AUV. The red dot represents the ASV static position while the blue line represents the AUV navigation during the first test execution.

In this section, we describe the experiments conducted to analyze the impact of the coordination strategy on the quality of the acoustic communication between the ASV and the AUV. In all experiments, the AUV must perform a transect of 160 m long by 5 m wide represented in Figure 15. Both missions were located in coastal waters with a depth between 7 to 20 m. The main mission and navigation parameters of the AUV are summarized in Table 4.

Two sets of tests have been performed with the test bench and parameters above-described:

1. Test without using any coordination strategy between the ASV and the AUV.
2. Test using the MMRS tracking strategy described above.

The results show how the ACL is affected by the distance between the acoustic receiver, placed on the AUV, and the transducer placed on the ASV. Next, the results of each set of experiments are described in detail.

1. Tests without tracking: To begin with, the first test consisted of mooring the ASV in a static position, using the keep position navigation strategy while the AUV executes the test bench mission.

**Figure 16:**
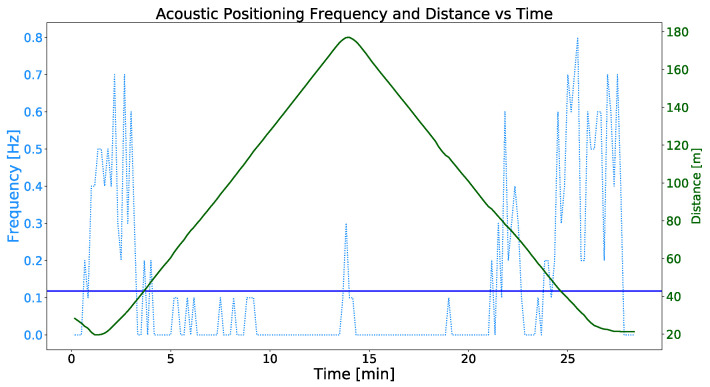
The green line represents the evolution over time of the distance between the ASV and the AUV while the latter performs the test bench mission template. The blue dotted line represents the frequency of the acoustic positioning corrections. Finally the blue line represents the mean of the acoustic positioning reception frequencies. The sample mean value of the acoustic positioning frequency is 0.117 Hz with a standard deviation of 0.198.

The results obtained from this experiment are shown in Figure 16. As can be seen, the frequency of the ACL positioning messages ranges from 0.2 Hz to 0.7 Hz when the distance between the vehicles is between 20 and 80 m and drops drastically to values between 0 Hz and 0.1 Hz when this distance is exceeded. The mean frequency of these messages is 0.1176 Hz. Then, it can be stated that the frequency of reception of the acoustic positioning strongly depends on the distance between the transducer and the receiver, among other factors. It should be noticed the anomalous acoustic positioning frequencies received at the end of the transect, just when the relative orientation between the ASV and the AUV changed. During this period, focused around minute 13, the acoustic positioning received frequencies rises to 0.3 Hz, a three times higher value than the average sampled frequency during the test.

2. Tests with tracking: In the second sea trial, the ASV was programmed to track the AUV while the latter performs the same mission as in the first test. The parameters for the tracking strategy used in this case are shown in Table 5.

**Figure 17:**
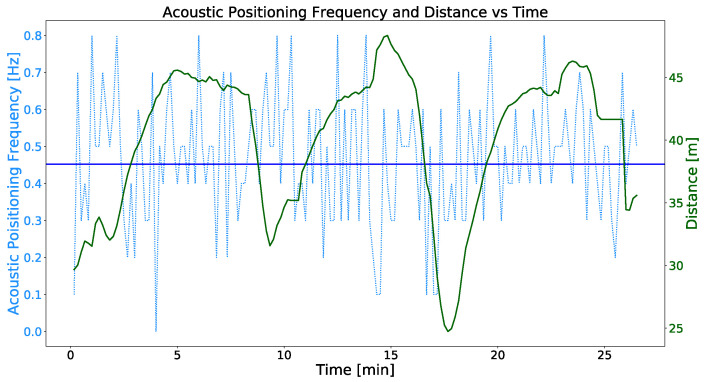
The green line represents the time evolution of the distance between the ASV and the AUV while the latter performs the test bench mission template and the ASV runs the tracking coordination strategy. The blue dotted line represents the frequency of the acoustic positioning corrections. Finally, the blue line represents the mean of the acoustic positioning reception frequencies. The sample mean value of the acoustic positioning frequency is 0.468 Hz.

Figure 17 shows the same information as Figure 16, the acoustic positioning frequency and distance between both vehicles versus the time. In this case, by applying the tracking coordination strategy, the distance between the ASV and the AUV has been significantly reduced to a range between 25 and 47 m compared to the experiments without tracking. As for the frequency of the acoustic positioning corrections, the values range between 0.1 Hz and 0.8 Hz, obtaining a sample mean frequency of 0.468 Hz and a standard deviation of 0.169.

Having analyzed the results of the acoustic communication tests, we can state that the use of the MMRS tracking coordination strategy provides substantial advantages. The significant distance reduction between the acoustic transponder and transducer by applying the tracking strategy results in a significant increase of the mean acoustic positioning frequency from 0.117 Hz to 0.468 Hz. Then, it can be stated that, applying the tracking coordination strategy, the mean ACL positioning reception frequency is about four times higher increasing from 0.117 Hz to 0.468 Hz.

## 8. Conclusions and Future Work

The new ASV Xiroi II has been presented as a fully operational vehicle with reliable hardware and accurate software that allows a series of sea trials to be carried out with satisfactory results. The main objectives of the Xiroi II have been reached.

- The software, an adapted version of the ROS-based COLA2 architecture, has been presented and detailed to serve as a reference for future ASV developments.
- With the new architecture, the vehicle has the ability to apply a variety of precise navigation strategies such as goto, keep position, section, or mission. This navigation behaviors are useful for the ASV to act as a reliable observation platform for shallow water benthic habitats. All these behaviors has been described, adjusted, and tested in real operating conditions with optimum results in both position and velocity control tests.
- Regarding the MMRS coordination strategies, an additional navigation behavior has been implemented for the tracking of an AUV. The analysis of the navigation frequency and acoustic communication tests shows that the designed tracking strategy achieves a significant increase from 0.177 Hz to 0.468 Hz in the reception frequency of the acoustic positioning corrections. This improvement allows the AUV to receive the acoustic positioning more frequently, which makes the vehicle localization more accurate.

In the light of these results, there are some challenging aspects to add and to improve. For the time being, we focus on the following issues:

- A new buoyancy hull design should be done taking into account the lightness, hydrodynamic, and modularity that characterizes the present vehicle. In addition, another frame should be built to rise the electronics case above the sea surface and prevent splashing.
- In terms of coordination strategies, new algorithms can be implemented to further improve the ACL communication, paying special attention to the relative orientation between vehicles as this factor seems to have some residual impact on acoustic reception.
- In terms of ACL, an analysis should be conducted to determine the improvement on the AUV localization when using the MMRS coordination strategy.
- In terms of MMRS coordination strategies, expand the tracking strategy with the aim of improving the acoustic communication link to more than two marine vehicles.

## Figures and Tables

**Table 1:** Main components and specifications for Xiroi II ASV.

Vehicle Specifications	
Battery	Lithium-Ion, 14.8 v 100 Ah
Actuators	2 × Blue Robotics T200
Weight	45.8 kg
Payload capacity	20 kg
Dimensions	1.75 × 1 × 0.4 m
Computer	Intel Core i7
Software	Ubuntu 18.04 and ROS Melodic

**Table 2:** Battery pack specifications.

Battery Pack Specifications	
Rechargeable cells	Li-ion Samsung ICR18650-26F
Voltage	14.8 V
Capacity	100 Ah
Power	1480 W
Weight	7.2 Kg

**Table 3:** Computer components and specifications.

Computer Specifications	
Motherboard	MSI MPG Z390I
	Gaming Edge AC
RAM memory	2 × 8 GB
SSD storage disk	240 GB
HDD storage disk	1 TB
Processor	INTEL CORE i7

**Table 4:** Test bench mission parameters.

Test Bench Mission Parameters	
AUV velocity	0.3 [m/s]
AUV altitude	5 [m]
Mission length	160 [m]
Mission width	5 [m]
ACL power	6 [dB]

**Table 5:** Tracking parameters.

Tracking Parameters	
Tracking radius	65 [m]
Adrift radius	45 [m]
Repulsion Radius	25 [m]

## Data Availability

All data and custom code support this study are available from the author A.M.-T., upon reasonable request.
